# Prevalence of Anemia and Its Associated Factors Among Schoolchildren in Metu Town Attending Public Primary Schools, Southwest Ethiopia

**DOI:** 10.1002/hsr2.72682

**Published:** 2026-06-22

**Authors:** Lemi Ushu Sime, Tilahun Yamane, Wakjira Kebede

**Affiliations:** ^1^ Department of Medical Laboratory Science, College of Health Science Metu University Metu Ethiopia; ^2^ School of Medical Laboratory Sciences, Institute of Health Jimma University Jimma Ethiopia

**Keywords:** anemia, associate factor, ethiopia, schoolchildren

## Abstract

**Background:**

Anemia is a worldwide public health problem, affecting more than half of schoolchildren in developing countries. The prevalence of anemia has been studied in various communities in Ethiopia. However, data on schoolchildren in Mattu town have been lacking.

**Objective:**

This study aimed to determine the prevalence and associated factors of anemia among schoolchildren attending a public primary school in Mattu town, southwest Ethiopia.

**Methods:**

A school‐based cross‐sectional study recruited 317 children aged 7–14 years in 2024. Sociodemographic and socioeconomic data were collected via questionnaires. Blood analyses were performed for complete blood count, red blood cell morphology, malaria, and intestinal parasites. Data analysis was conducted using SPSS version 23, with logistic regression used to identify factors associated with anemia at *p* value < 0.05.

**Result:**

A total of 330 schoolchildren were included in the study, with a 96.1% response rate. The overall prevalence of anemia in this study was 33 (10.4%, 95%CI: 7.27%, 14.31%). Mild anemia was found in 69.7% of cases, and females comprised 53.3% of participants, with a mean age of 11.1 years (SD ± 1.798). The factors significantly associated with anemia included mothers with no formal education (AOR = 5.02, 95% CI: 1.75–14.39), male gender ((AOR = 3.15, 95% CI: 1.20–8.28)), low family income (AOR = 4.71, 95% CI: 1.75–12.68), intestinal parasitic infection (AOR = 4.48, 95% CI: 1.77–11.37), and a habit of consuming coffee or tea with meals (AOR = 3.96, 95% CI: 1.55–10.11).

**Conclusion:**

This study found a mild prevalence of anemia among schoolchildren in Mattu town. Anemia was associated with low maternal education, low family income, male sex, and intestinal parasitic infections. Targeted interventions such as health education, deworming programs, and awareness of tea/coffee consumption with meals are recommended to improve child health outcomes.

**Abbreviations**
AOR: adjusted odds ratio
CBC: complete blood count
CI: confidence interval
COR: crude odds ratio
EDTA: ethylenediamine tetraacetic acid
Hb: hemoglobin
IPI: intestinal parasite infection
MCH: mean corpuscular hemoglobin
MCHC: mean corpuscular hemoglobin concentration
SD: standard deviation
SPSS: Statistical Package for Social Science
WHO: World Health Organization

## 1 Introduction

In 2022, anemia affected approximately 1.93 billion people worldwide, accounting for about 27% of the global population. The burden remains disproportionately higher in developing countries, where prevalence can reach up to 89% [1]. Globally, in 2019, anemia had a prevalence of 22.8% and contributed to 58.6 million people living with disabilities. The burden was highest in African countries, indicating a disproportionate impact on the region [2]. A survey study illustrates that anemia is a significant public health problem worldwide, with low‐income countries in Western and Central Sub‐Saharan Africa and South Asia being disproportionately affected. Studies conducted in these regions show a high prevalence of anemia in schoolchildren, ranging from 40% to 60% [3]. Anemia imposes a substantial burden on schoolchildren by negatively affecting academic performance and increasing school absenteeism [4]. Anemia affects a substantial proportion of schoolchildren in developing countries, with a reported prevalence of 79.6% [5]. According to the Ethiopian National Micronutrient Survey, the prevalence of anemia among schoolchildren was 25.8% after adjustment for altitude [6]. Similarly, in Ethiopia, schoolchildren suffering from anemia with mild to severe public health problems based on WHO criteria, extending from 7.6% [7] to 43.7% [8]. These studies highlight the adverse effects of anemia on schoolchildren, including impaired learning, physical growth, cognitive development, and increased susceptibility to infections. Several studies have indicated that schoolchildren attending government schools have a higher rate of anemia than those attending private schools [9]. Anemia is a condition characterized by a reduced erythrocyte count or hemoglobin level, resulting in decreased oxygen‐carrying capacity of the blood [10], The severity of anemia among schoolchildren aged 6–14 years is classified based on adjusted hemoglobin (Hb) concentration as mild (10.0–11.9 g/dL), moderate (7.0–9.9 g/dL), and severe (< 7.0 g/dL) [11]. Based on prevalence, anemia is classified as a mild public health problem when it affects 5.0–19.9% of the population, a moderate public health problem at 20.0–39.9%, and a severe public health problem when the prevalence is 40% or higher [12]. Iron deficiency is the leading cause of anemia among schoolchildren, impairing oxygen transport throughout the body and affecting neurotransmitter synthesis [13, 14]. Other contributing factors, including deficiencies in folate, vitamin B12, and vitamin A, parasitic infections, and impaired red blood cell production, further worsen the condition. Consequently, anemia negatively affects the physical growth, cognitive performance, and learning capacity of schoolchildren [11, 15]. Overall, the pathophysiology of anemia differs according to its underlying causes [16]. Although previous studies in the local area have assessed the prevalence of anemia and its associated factors, limited attention has been given to the morphological classification of anemia, and evidence specifically among primary schoolchildren in Mattu town, southwest Ethiopia, remains scarce. This gap in context‐specific data may hinder the development of effective interventions to address childhood anemia in the area. Therefore, this study aimed to assess the prevalence of anemia and its associated factors among public primary schoolchildren in Mattu town.

## 2 Materials and Methods

### 2.1 Study Design, Setting, and Population

A school‐based cross‐sectional study was conducted among schoolchildren aged 7–14 years attending a public primary school in Mattu Town, Ethiopia. Mattu is located in the Oromia Region, approximately 600 km southwest of the capital city, Addis Ababa. The town is situated at an elevation of 1605 m above sea level. In 2021, Mattu had an estimated population of 41,231, of whom 21,027 were men, and 20,204 were women [17]. According to the town's Education Office, there were 12 primary schools in the town. This study focused on the seven public primary schools, which had a total student population of 5375 (Source: Mattu Town Education Bureau, 2024). The study participants were schoolchildren aged 7–14 years whose parents or guardians provided consent and who were willing to participate. Schoolchildren who had received anti‐helminthic medication or anemia treatment within the 3 months preceding data collection were excluded from the study.

### 2.2 Sample Size Calculation

To ensure a representative sample and precise estimation of anemia prevalence among schoolchildren, a single population proportion formula was used to determine the required sample size. This calculation was based on a previously reported anemia prevalence of 15.4% among schoolchildren in Yirgacheffe, South Ethiopia [18]. A desired margin of error of 5% and a 95% confidence level were used to ensure reliable generalizability of the findings. Additionally, a design effect of 1.5 was incorporated to account for potential clustering of data within schools. Finally, assuming a 10% non‐response rate, the formula yielded a minimum sample size of 330 schoolchildren.

$$n=Z\alpha /2^{2}p\left(1-p\right)/d^{2}$$

$$n={\left(1.96\right)}^{2}\times 0.154\left(1-0.154\right)/{\left(0.05\right)}^{2}=200$$

200×105=300

After the non‐response rate of 10% was added, the total sample size was 300 + 30 = 330. Therefore, 330 schoolchildren were selected for the study.

### 2.3 Sampling Techniques

A multistage sampling technique was employed to select study participants. In the first stage, all seven public primary schools in Mattu Town were listed, and three schools were selected using simple random sampling (lottery method), giving each school an equal chance of inclusion and minimizing selection bias. In the second stage, the number of children aged 7–14 years in each selected school was obtained from the school directors, and the total sample size (*n* = 330) was proportionally allocated based on the size of the target population in each school. In the third stage, participants were selected using systematic random sampling from class rosters. The sampling interval was determined by dividing the total eligible population by the allocated sample size (*K* = 3586/330 ≈ 11). A random starting point between 1 and *K* was selected by lottery, and every *K*th student was included until the required sample size for each school was reached, beginning with Grade 1 in all selected schools.

**Figure 1:**
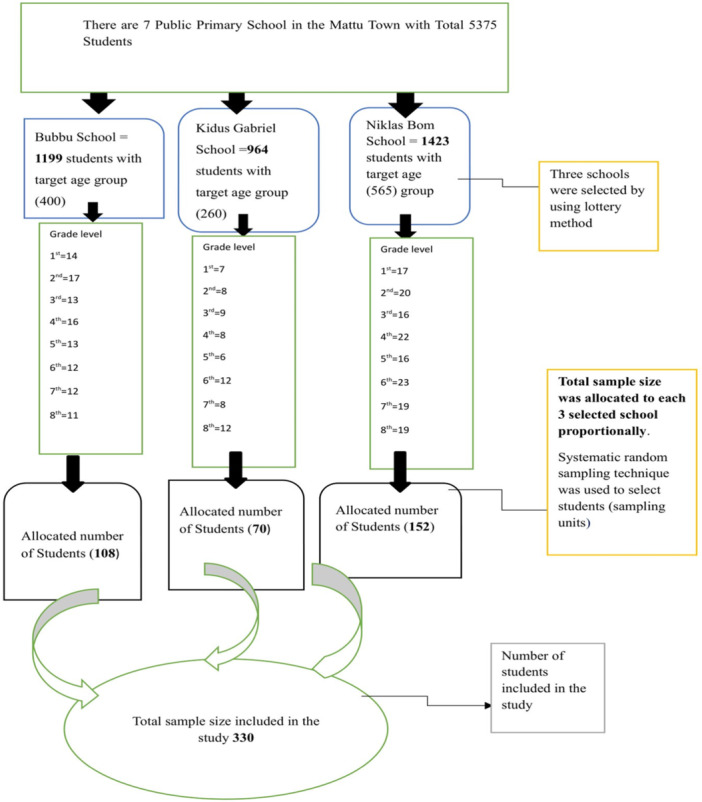
Schematic diagram indicating sampling technique among schoolchildren in Mattu town, attending public primary schools in southwest Ethiopia, 2024.

The sampling frame and distribution were based on the target age group (7–14 years) rather than the total school enrollment. Specifically, Bubbu School had 400 eligible children, Kidus Gabriel School had 260, and Niklas Bom School had 565. In contrast, the larger figures (1199, 964, and 1423, respectively) represented the total student populations across all grades. The figure referenced illustrates only the distribution of the target age group within each selected school, not the total school enrollment (Figure 1).

### 2.4 Data Collection Tools and Methods

#### 2.4.1 Sociodemographic and Economic Data Collection

Sociodemographic and socioeconomic data were collected from the legal guardians of participating schoolchildren via face‐to‐face interviews. A structured questionnaire was developed through adaptation from relevant literature and previously validated instruments [19, 20, 21], and it was translated into Afan Oromo, the local language. Back‐translation to English ensured consistency and accuracy. School directors and/or class teachers facilitated contact with parents and guardians.

#### 2.4.2 Blood Sample Collection

Approximately 3 mL of venous blood was collected from each participant using a sterile technique into an EDTA (ethylenediaminetetraacetic acid) tube. This blood was used for both a complete blood count (CBC) analysis and a peripheral blood smear examination for red blood cell morphology. The Advia560 hematology analyzer (Siemens Laboratories Diagnostics Division, Germany) was employed for CBC analysis, utilizing a combination of impedance, photometric light absorbance, optical light scatter, and diffraction methods. Blood specimens were transported upright and secured within a screw‐cap container to minimize temperature changes and agitation. Analysis occurred within 4 h of collection, as recommended by the reference guideline [22, 23]. Two thin blood smears were prepared, stained with Wright's stain, and examined for red blood cell morphology. Each slide was observed by one hematologist and a senior laboratory technologist. Anemia type was determined by morphological examination of peripheral blood films, with results compared to mean corpuscular volume (MCV), mean corpuscular hemoglobin concentration (MCHC), and red cell distribution width (RDW) values obtained from the automated analyzer.

#### 2.4.3 Blood Film Preparation for Malaria Diagnosis

One to two drops of blood were collected and used to prepare thin and thick blood films on separate clean, frosted slides. The thick film enhances sensitivity for detecting low parasite densities. The thin film was methanol‐fixed and stained with diluted Giemsa stain (10%) at a pH of 7.2 using buffered water to highlight parasite inclusions within red blood cells, facilitating morphological identification of parasite species. Each slide was examined by two experienced laboratory technologists.

#### 2.4.4 Stool Sample Collection and Examination for Intestinal Parasite

The children were provided with clean, labeled, leak‐proof, dry, wide‐mouthed cups, an applicator stick, and toilet tissue paper. Disposable tissue paper was used to reduce contamination. They were also instructed to tightly close the cup immediately after placing the sample into the container. They were directed to collect approximately 4 g of stool into the cups. Stool specimens were transported in screw‐capped containers containing 10% formalin to the laboratory for immediate processing. Microscopic examination was performed using both direct saline wet mount preparation and formal ether concentration techniques.

#### 2.4.5 Quality of Data

The quality of the data was ensured through strict adherence to standard operating procedures (SOPs) throughout specimen collection, processing, and all laboratory procedures. Stained blood smears were evaluated macroscopically and microscopically; initial screening was performed using low‐power magnification (10×) to assess overall smear quality and to examine the edges and central area of the slide for clumping or artefacts. The quality of Wright stain was assessed using thin blood films stained at different staining times by comparing the staining characteristics of red and white blood cells. Giemsa stain quality was ensured through daily preparation of fresh working solution and filtration immediately before use to maintain staining consistency. Stool samples processed using the formalin–ether concentration technique were examined under light microscopy using 10× and 40× objectives, and all negative and positive slides were independently rechecked by a senior laboratory technologist to ensure accuracy and reduce observer bias. Laboratory findings were systematically recorded using participant identification numbers to maintain traceability and minimize recording errors. To further ensure data quality, data collectors received standardized training prior to data collection to reduce technical and observational bias. A pre‐test was conducted on 5% of the total sample size to assess the clarity, validity, reliability, and acceptability of the questionnaire, and appropriate modifications were made based on the findings. In addition, continuous supervision was provided during data collection. Finally, all collected data were checked, cleaned, and verified before analysis to identify inconsistencies, missing values, and outliers. Detected errors were corrected promptly to ensure completeness and accuracy of the final dataset.

#### 2.4.6 Data Analysis and Interpretation

Data entry was performed using EpiData statistical software version 4.6.02. Statistical analysis was then conducted using SPSS version 23. Descriptive statistics, including means, frequencies, percentages, and cross‐tabulations, were used to characterize participants in relation to relevant variables. All tests of significance were two‐sided. A logistic regression model was constructed to identify factors associated with anemia. The Hosmer–Lemeshow test was used to assess model goodness‐of‐fit, and multicollinearity was evaluated using variance inflation factor (VIF) analysis. The normality of continuous variables was assessed visually using histograms. Variables with a *p* value < 0.25 in the bivariate analysis were considered candidates for inclusion in the final multivariable logistic regression model. Odds ratios (OR) with corresponding 95% confidence intervals (CI) were calculated to measure the association between participant characteristics and anemia. A *p* value ≤ 0.05 was considered statistically significant. The results were disseminated to the Mattu Town Health Office and the Ilu Aba Bor Zonal Health Office.

#### 2.4.7 Ethical Consideration

The study was approved by the ethical review board of the Institute of Health, Jimma University (reference number IHRPGn/448). A letter of support was obtained from the Mattu town health and education office. Official permission was submitted to each school director. Written informed consent was obtained from the parents or legal guardians of the children, and an additional child assent form was obtained from schoolchildren. Anyone who was not willing to participate was excluded from the study. Confidentiality of results was strictly maintained. Confidentiality was maintained by the numeric coding of samples and questionnaires. Children with parasitic infections and those with anemic results were treated free of charge.

## 3 Results

### 3.1 Characteristics of the Participants

A total of 330 schoolchildren were enrolled in the study. Of these, 3.9% declined laboratory testing and were excluded, resulting in a final sample size of 317 participants. The majority of participants were female (53.3%, *n *= 169). Approximately half of the participants (50.5%, *n *= 160) were in the 7–10‐year age group. The mean age of the children was 11.1 years (SD ± 1.798), while the mean age of caregivers was 42.08 years (SD ± 10.772). A substantial proportion of parents (42.6%, *n *= 135) and fathers (67.8%, *n *= 215) had attained a diploma or higher level of education. High monthly income was reported by 78.5% (*n *= 249) of parents (Table 1).

**Table 1:** Sociodemographic and other selected variables of schoolchildren in Mattu Town, attending public primary schools, southwest Ethiopia, 2024 (*N* = 317).

Variables	Category	Frequency	Percentage
Age	7–10	160	50.5
	11–14	157	49.5
Sex	Male	148	46.7
	Female	169	53.3
Grade level (child)	1–4	181	57.1
	5–8	136	42.9
Family size	≤ 5	190	59.9
	> 5	127	40.1
Mother's educational status	No formal education	64	20.2
	Primary school	56	17.7
	Secondary school	62	19.6
	Diploma and above	135	42.6
Mother's occupation	Employed*	141	44.5
	Housewife	41	12.9
	Merchant	100	31.6
	Daily laborer	35	11.0
Father's educational status	No formal education	28	8.8
	Primary school*	43	13.6
	Secondary school*	31	9.8
	Diploma and above	215	67.8
Father's occupation	Employed*	135	42.6
	Merchant	67	21.1
	Daily laborer	83	26.2
	Farmer	32	10.1
Monthly family income (ETB)	< 2000	32	10.1
	2000–3000	36	11.4
	> 3000	249	78.5
Child–guardian relationship	Mother	191	60.3
	Father	100	31.5
	Relatives*	26	8.2
Mother's marital status	Unmarried	23	7.3
	Married	272	85.8
	Separated*	22	6.9
Maternal or guardian age	≤ 34	131	41.3
	35–44	79	24.9
	≥ 45	107	33.8
Tea or coffee drinking habit with meal	Yes	57	18
	No	260	82
*Ascaris lumbricoides*	Positive	**30**	9.5
	Negative	287	90.5
Hook worm	Positive	**15**	4.7
	Negative	302	95.3
*Trichuris trichiura*	Positive	**8**	2.5
	Negative	309	97.5
Malaria infection	Positive	1	0.3
	Negative	316	99.7

*Note:* Separated* = divorced or widowed, employed* = both government and private, relatives* = any legal guardians other than mother and father. Bold values indicates statistically significant.

### 3.2 Prevalence of Anemia and Related Characteristics

In this study, the overall prevalence of anemia among schoolchildren was 10.4% (95% CI: 7.27–14.31). Among these, 53 children (16.7%) were diagnosed with intestinal parasitic infections. The prevalence of anemia among children infected with these parasites was 33.3% (10/30) for *Ascaris lumbricoides*, 46.7% (7/15) for hookworm, and 37.5% (3/8) for *Trichuris trichiura*. No cases of anemia were observed among children with malaria parasite infection. The prevalence of anemia was significantly higher among males (17.6%, *n *= 26) than females (4.1%, *n *= 7). A higher prevalence was also observed among children aged 7–10 years (14.4%, *n *= 23). Furthermore, anemia was more common among children from households with more than five family members (16.5%, *n *= 21), those whose mothers had no formal education (26.6%, *n *= 17), and those from low‐income households earning less than 2000 ETB per month (37.5%, *n *= 12) (Table 2).

**Table 2:** Prevalence of anemia among schoolchildren by socio‐demographic factors in Mettu town attending public primary schools, southwest Ethiopia, 2024 (*N* = 317).

Variables	Categories	Anemia	
		Anemic (%)	Non‐anemic (%)
Age	7–10	23 (14.4)	137 (85.6)
	11–14	10 (6.4)	147 (93.6)
Sex	Female	7 (4.1)	162 (95.9)
	Male	26 (17.6)	122 (82.4)
Grade level (child)	1–4	23 (12.7)	158 (87.3)
	5–8	10 (7.4)	126 (92.6)
Number of family members	≤ 5	12 (6.3)	178 (93.7)
	> 5	21 (16.5)	106 (83.5)
Mother's education	No formal education	17 (26.6)	47 (73.4)
	Primary school	2 (3.6)	54 (96.4)
	Secondary school	5 (8.1)	57 (91.9)
	Diploma and above	9 (6.7)	126 (93.3)
Mother's occupation	Employed	12 (8.5)	129 (91.5)
	House wife	5 (12.2)	36 (87.8)
	Merchant	12 (12.0)	88 (88.0)
	Daily laborer	4 (11.4)	31 (88.6)
Father's educational status	No formal education	3 (10.7)	25 (89.3)
	Primary school	4 (9.3)	39 (90.7)
	Secondary school	5 (16.1)	26 (83.9)
	Diploma and above	21 (9.8)	194 (90.2)
Father's occupation	Employed	15 (11.1)	120 (88.9)
	Merchant	5 (7.5)	62 (92.5)
	Daily laborer	8 (9.6)	75 (90.4)
	Farmer	5 (15.6)	27 (84.4)
Monthly family income (ETB)	< 2000	12 (37.5)	20 (62.5)
	2000–3000	6 (16.7)	30 (83.3)
	> 3000	15 (6.0)	234 (94.0)
Child–guardian relationship	Mother	21 (11.0)	170 (89.0)
	Father	11 (11.0)	89 (89.0)
	Relatives	1 (3.8)	25 (96.2)
Mother's marital status	Unmarried	3 (13.0)	20 (87.0)
	Married	28 (10.3)	244 (89.7)
	Separated	2 (9.1)	20 (90.9)
Maternal or guardian age	≤ 34	15 (11.5)	116 (88.5)
	35–44	7 (8.9)	72 (89.7)
	≥ 45	11 (10.3)	96 (30.3)
Habit of regular drinking tea or coffee with breakfast	Yes	13 (22.8)	44 (77.2)
	No	20 (7.7)	240 (92.3)
*Ascaris lumbricoides*	Positive	10 (33.3)	20 (66.7)
	Negative	23 (8.0)	264 (92.0)
Hook worm	Positive	7 (46.7)	8 (53.3)
	Negative	26 (8.6)	276 (91.4)
*Trichiura Trichuris*	Positive	3 (37.5)	5 (62.5)
	Negative	30 (9.7)	279 (90.3)
Malaria infection	Positive	—	1 (0.4)
	Negative	33 (10.4)	283 (99.6)

### 3.3 Factors Associated With Anemia Infection

To identify factors independently associated with anemia among primary schoolchildren, a multivariable logistic regression analysis was performed. Variables with a *p* value ≤ 0.25 in the bivariate analysis were included in the final model. After adjusting for potential confounders, maternal educational status, sex, monthly family income, intestinal parasitic infection, and regular consumption of coffee or tea with meals remained significantly associated with anemia. Children whose mothers had no formal education were more likely to be anemic (AOR = 5.02, 95% CI: 1.75–14.39). Likewise, male children (AOR = 3.15, 95% CI: 1.20–8.28), those from low‐income households (AOR = 4.71, 95% CI: 1.77–11.37), children with intestinal parasitic infections (AOR = 4.48, 95% CI: 1.77–11.37), and those who regularly consumed coffee or tea with meals (AOR = 3.96, 95% CI: 1.55–10.11) had significantly higher odds of anemia (Table 3).

**Table 3:** Bivariate and multivariable logistic regression analysis of selected variables associated with anemia among schoolchildren in Mattu town, attending public primary schools, southwest Ethiopia, 2024 (*n* = 317).

Variables	Categories	Anemia		COR (95%CL)	*p* value	AOR (95%CL)	*p* value
		Anemic (%)	Non anemic (%)				
Age (year)	7–10	23 (14.4)	137 (85.6)	2.45 (1.13, 5.37)	**0.023**	2.06 (0.82, 5.18)	0.126
	11–14	10 (6.4)	147 (93.6)	1.00		1.00	
Sex	Female	7 (4.1)	162 (95.9)	1.00		1.00	
	Male	26 (17.6)	122 (82.4)	4.93 (2.07,11.74)	**0.000**	3.15 (1.20,8.28)	**0.02** *
Grade level (child)	1–4	23 (12.7)	158 (87.3)	4.93 (2.07, 11.74)	**0.000**	1.21 (0.41, 3.61)	0.732
	5–8	10 (7.4)	126 (92.6)	1.00		1.00	
Number of family members.	< 5	12 (6.3)	178 (93.7)	1.00		1.00	
	> 5	21 (16.6)	106 (83.5)	2.94 (1.39, 6.21)	**0.005**	1.46 (0.60, 3.54)	0.405
Mother's education	No formal education	17 (26.6)	47 (73.4)	5.06 (2.11,12.14)	**0.000**	5.02 (1.75,14.39)	**0.003** *
	Primary school	2 (3.6)	54 (96.4)	0.52 (0.12,2.48)	0.411	0.60 (0.11,3.23)	0.553
	Secondary school	5 (8.1)	57 (91.9)	1.23 (0.39,3.83)	0.723	2.24 (0.59,8.46)	0.236
	Diploma and above	9 (6.7)	126 (93.3)	1.00		1.00	
Monthly family income	< 2000	12 (37.5)	20 (62.5)	9.36 (3.86,22.70)	**0.000**	4.71 (1.75,12.68)	**0.002** *
	2000–3000	6 (16.7)	30 (83.3)	3.12 (1.13,8.65)	**0.029**	2.14 (0.65,7.06)	0.21
	> 3000	15 (6.0)	234 (94.0)	1.00		1.00	
Tea or coffee drinking habit	Positive	13 (22.8)	44 (77.2)	3.55 (1.64,7.65)	**0.001**	3.96 (1.55,10.11)	**0.004** *
	Negative	20 (7.7)	240 (92.3)	1.00		1.00	
IpI	Positive	13 (24.5)	40 (75.5)	3.97 (1.83,8.60)	**0.000**	4.48 (1.77,11.37)	**0.002** *
	Negative	20 (7.6)	244 (92.4)	1.00		1.00	

*Note:* Bold values indicates statistically significant.

* Variables that are significant in multivariable analysis.

## 4 Discussion

This study investigated the prevalence of anemia and its associated factors among schoolchildren in Mattu, Ethiopia. The overall prevalence of anemia (10.4%) is classified as a mild public health problem according to WHO criteria [24]. This suggests that anemia is a public health concern, but not a severe problem in this area. The finding is consistent with a study conducted in Durbite Town, northwest Ethiopia (10.7%) [25], in Mekelle (11%) [26], Uganda, 11.8% [27], Vietnam 12.9% [28], China 11.7% [29], Mexico, 12% [30], in Brazil 9.3% [19], and Serbia (10.8%) [31]. However, it was lower than earlier reports from Jimma itself and other regions within Ethiopia, including Jimma (37.6–43.7)% [8, 32], Arba Minch 37.3% [15], Benishangul Gumuz 33.9% [33], Gondar 15.5% [21], Somali filtu town 23.66% [34], Afar 22.8% [35], and Kersa 27.1% [36]. These discrepancies likely reflect variations in study periods, locations, socioeconomic factors, and disease burdens (e.g., malaria).

Similarly, the prevalence of anemia was lower than studies done in African and other countries, such as Egypt (32.8%, 38.7%, and 53.1% [37, 38, 39], Ghana 50.3% [40], Guinea 85% [41], Sudan 29.7% [42], democratic republic of Congo 41.1% [43], Yemen 48.7% [44], Nepal 37.9% [45], Pakistan 64.6% [46], and Serbia 18% [47]. The discrepancy may be attributed to the differences in study settings (some were carried out in rural areas). In addition, the consumption of iron‐rich foods, for example, teff, the iron‐rich staple food in Ethiopia, might have contributed to the low prevalence of anemia in the study area. But compared to other studies, our finding was higher than the study findings from northwest Ethiopia (7.6%) [7], Addis Ababa (5.83%) [48], and Cameroon 5% [49].

Several factors were significantly associated with anemia. Children whose mothers had no formal education were more likely to be anemic compared to those whose mothers had a diploma or higher education level (AOR = 5.02, 95% CI: 1.75–14.39). This finding is consistent with a study conducted in Jimma, Ethiopia [32], Benishangul‐Gumuz Pawe Town, Metekel Zone [33], and Yemen [44], highlighting the importance of maternal education for promoting child health practices. Uneducated mothers may not understand the nutritional requirements for children and follow the recommended child feeding practices [50]. Furthermore, maternal education is a key determinant of child health, as it influences knowledge, attitudes, and practices related to nutrition, hygiene, and healthcare. Mothers with no formal education may have limited awareness of age‐appropriate feeding practices, dietary diversity, and the importance of micronutrients, leading to inadequate nutritional intake among children. In addition, maternal education affects health‐seeking behaviors, including timely use of healthcare services, adherence to vaccination schedules, and prompt treatment of infections that may contribute to anemia. Educated mothers are also more likely to adopt proper hygiene practices and to understand health information, which supports better child care and nutrition. Consequently, children of uneducated mothers are at higher risk of anemia and other nutrition‐related disorders.

Similarly, children from families with lower monthly income were more likely to be anemic compared to those from higher‐income households (AOR = 4.71, 95% CI: 1.75–12.68), suggesting an association between socioeconomic status and access to nutritious foods. This finding is consistent with a study conducted in Jimma, Ethiopia [8, 32], Somali filtu town [34], and Yemen [44].

Intestinal parasitic infections were also significantly associated with anemia. Children with IPIs were more likely to be anemic compared to their counterparts (AOR = 4.48, 95% CI: 1.77–11.37), likely due to blood loss and impaired nutrient absorption caused by the parasites [18, 51]. This study is similar to the one conducted in Benishangul‐Gumuz Pawe Town, Metekel Zone [33], Jimma [8], Somali filtu town [34], Arba Minch [15], and Gondar town [21].

This study also found that male children were more likely to be anemic than females (AOR = 3.15, 95% CI: 1.20–8.28). This may be explained by the fact that boys are often more engaged in outdoor and physically active play, which increases their exposure to soil‐transmitted infections, a known risk factor for anemia. These activities may also increase energy and micronutrient requirements, and when these needs are not met, the risk of anemia may be further elevated. This finding is consistent with a study conducted in Durbite Town, northwest Ethiopia [25]. However, some studies have reported the opposite trend [9]. This may be because some females start to see menstrual cycles at this age. Finally, regular consumption of tea or coffee with meals was associated with a higher prevalence of anemia (AOR = 3.96, 95%; 1.55, 10.11). This is likely due to the inhibitory effect of tea and coffee on iron absorption. This study corresponds with a study conducted in India [52] and Gondar [53].

### 4.1 Limitation

A cross‐sectional study design restricts the ability to determine temporal or causal relationships between anemia and its associated factors, as exposure and outcome were assessed at the same time. Therefore, it is not possible to confirm whether the identified factors preceded or resulted from anemia. In addition, the study did not include a detailed assessment of children's dietary intake, which may have led to residual confounding related to nutritional deficiencies. Furthermore, inflammatory conditions such as subclinical infections, which can influence hemoglobin levels through anemia of inflammation, were not screened. These unmeasured factors may have affected the observed prevalence and associated factors of anemia.

## 5 Conclusion

Anemia remains a significant public health concern among schoolchildren in Mattu town. It is associated with multiple factors, including maternal education, socioeconomic status, intestinal parasitic infections, sex, and dietary practices. These findings highlight the need for integrated nutrition and infection control strategies. Future studies should employ longitudinal or interventional designs to better establish causal relationships and evaluate the effectiveness of targeted anemia prevention strategies in this population.

## Author Contributions

**Lemi Ushu Sime:** conceptualization, methodology, software, data curation, investigation, validation, formal analysis, supervision, funding acquisition, visualization, project administration, resources, writing – original draft, writing – review and editing. **Tilahun Yamane:** conceptualization, writing – original draft, writing – review and editing. **Wakjira Kebede:** conceptualization, data curation, writing – review and editing, writing – original draft, formal analysis, and methodology.

## Funding

The authors have nothing to report.

## Ethics Statement

The study was conducted in accordance with the ethical principles outlined in the Declaration of Helsinki. Ethical approval was obtained from the ethical review board of the Institute of Health, Jimma University (reference number: IHRPGn/448). A letter of support was secured from the Mattu Town Health and Education Office, and official permission was obtained from each participating school director. Written informed consent was obtained from the parents or legal guardians of the children, and assent was additionally obtained from the participating schoolchildren. Participation in the study was entirely voluntary, and individuals who were unwilling to participate were excluded from the study without any consequences. Confidentiality and privacy of participants were strictly maintained through the use of numeric coding for samples and questionnaires, with no personal identifiers recorded. Furthermore, children diagnosed with parasitic infections or anemia during the study were provided with appropriate treatment free of charge in accordance with ethical standards and beneficence principles.

## Conflicts of Interest

The authors declare no conflicts of interest.

## Transparency Statement

The corresponding author, Lemi Ushu Sime, affirms that this manuscript provides an honest, accurate, and transparent account of the work performed. All key aspects of the study are included, and any differences between the planned and actual study have been fully described.

## Data Availability

The data that support the findings of this study are available from the corresponding author upon reasonable request.
